# Comparison of the Equine Reference Sequence with Its Sanger Source Data and New Illumina Reads

**DOI:** 10.1371/journal.pone.0126852

**Published:** 2015-06-24

**Authors:** Jovan Rebolledo-Mendez, Matthew S. Hestand, Stephen J. Coleman, Zheng Zeng, Ludovic Orlando, James N. MacLeod, Ted Kalbfleisch

**Affiliations:** 1 Department of Biochemistry and Molecular Biology, School of Medicine, University of Louisville, Louisville, Kentucky, United States of America; 2 Maxwell H. Gluck Equine Research Center, Department of Veterinary Science, University of Kentucky, Lexington, Kentucky, United States of America; 3 Department of Computer Science, University of Kentucky, Lexington, Kentucky, United States of America; 4 Centre for GeoGenetics, Natural History Museum of Denmark, University of Copenhagen, Copenhagen, Denmark; 5 Intrepid Bioinformatics, Louisville, Kentucky, United States of America; Wageningen UR Livestock Research, NETHERLANDS

## Abstract

The reference assembly for the domestic horse, EquCab2, published in 2009, was built using approximately 30 million Sanger reads from a Thoroughbred mare named Twilight. Contiguity in the assembly was facilitated using nearly 315 thousand BAC end sequences from Twilight’s half brother Bravo. Since then, it has served as the foundation for many genome-wide analyses that include not only the modern horse, but ancient horses and other equid species as well. As data mapped to this reference has accumulated, consistent variation between mapped datasets and the reference, in terms of regions with no read coverage, single nucleotide variants, and small insertions/deletions have become apparent. In many cases, it is not clear whether these differences are the result of true sequence variation between the research subjects’ and Twilight’s genome or due to errors in the reference. EquCab2 is regarded as “The Twilight Assembly.” The objective of this study was to identify inconsistencies between the EquCab2 assembly and the source Twilight Sanger data used to build it. To that end, the original Sanger and BAC end reads have been mapped back to this equine reference and assessed with the addition of approximately 40X coverage of new Illumina Paired-End sequence data. The resulting mapped datasets identify those regions with low Sanger read coverage, as well as variation in genomic content that is not consistent with either the original Twilight Sanger data or the new genomic sequence data generated from Twilight on the Illumina platform. As the haploid EquCab2 reference assembly was created using Sanger reads derived largely from a single individual, the vast majority of variation detected in a mapped dataset comprised of those same Sanger reads should be heterozygous. In contrast, homozygous variations would represent either errors in the reference or contributions from Bravo's BAC end sequences. Our analysis identifies 720,843 homozygous discrepancies between new, high throughput genomic sequence data generated for Twilight and the EquCab2 reference assembly. Most of these represent errors in the assembly, while approximately 10,000 are demonstrated to be contributions from another horse. Other results are presented that include the binary alignment map file of the mapped Sanger reads, a list of variants identified as discrepancies between the source data and resulting reference, and a BED annotation file that lists the regions of the genome whose consensus was likely derived from low coverage alignments.

## Introduction

The current reference assembly for *Equus caballus*, EquCab2, was released in 2007 and published by Wade et al. in 2009[1]. It has served as the basis for many subsequent equine studies including whole genome variant detection and genotyping, RNA sequencing, epigenetic, and comparative genomics studies. The foundation of the reference was approximately 30 million Sanger reads produced on the ABI 3730 from a Thoroughbred mare named Twilight. Also included were approximately 315 thousand BAC end sequences derived from Twilight’s half brother named Bravo. Assembly of these reads produced a reference genome comprised of the 31 autosomal chromosomes, chromosome X, mitochondrial DNA (MT) and a collection of unincorporated contigs referred to as chrUn. In the time since EquCab2 was assembled, nearly every sequence read that has been generated for the domestic horse[2–4] or other equids[5] has been compared to this reference. As was inevitable, analyses of these additional datasets have revealed some inconsistencies. For example, several hundred variants discovered in mapped RNA-seq contigs relative to EquCab2 extend the reading frame of mRNAs in which they reside while concurrently being homozygous variants in Twilight genomic resequencing data[6]

The average coverage for the reference assembly was reported as 6.8X[1]. Genomic coverage in a shotgun sequencing effort is dictated by a Poisson process and will necessarily result in many regions of the genome covered by two or fewer reads. Regions with greater coverage benefit from high read depth which averages out sequencing errors inherent in Sanger reads, while those with less coverage will be more susceptible to retained errors. Further, some ambiguity is present within the equine research community as to whether the EquCab2 reference sequence is derived exclusively from Twilight. The goals of this work were to identify regions of the EquCab2 that are inconsistent with sequence data generated from Twilight, determine which of these inconsistencies can be explained as contributions from Bravo, and establish which can not be explained by either. In this study, the original Sanger data plus new Illumina short read data from Twilight were used, together with the original Bravo BAC-end Sanger reads. Here, we present the results of an alignment of the original 30 million Sanger reads plus new Illumina short read data from Twilight, and the 315 thousand from Bravo to the EquCab2 reference assembly they ultimately produced. The resulting mapped dataset identifies those regions with low Sanger read coverage, as well as variation in genomic content that is not consistent with either the original Twilight Sanger data or approximately 40X of new genomic sequence data generated from Twilight.

As a result of this analysis, we provide binary alignment map (BAM) files containing the mapped Twilight and Bravo Sanger reads, variant call format (VCF) files that list all variants measured for Twilight relative to EquCab2, a BED format annotation file that lists the regions of EquCab2 covered by two or fewer reads in the Twilight Sanger dataset, and finally, a companion 40X Illumina paired end dataset generated from Twilight’s genomic DNA. These files may be accessed for use in browsers with other equine sequence data or for incorporation in analysis pipelines.

## Results

### Read mapping

Twilight Sanger read mapping statistics are shown in Table 1. The Twilight reads, once trimmed for quality and vector, that were 200 bases or greater in length were mapped via the BWA-SW[7] module of the BWA[8] alignment program (described in Methods). In total, there were more than 28 million of these reads and 97.5% of them mapped to chr1-31, chrX, or MT collectively (1.1% mapped to chrUn). Those reads that were less than 200 bases in length after trimming were run through the standard BWA aln module (also described in Methods). The entire set of reads less than 200 and greater than or equal to 50 bases in length were mapped to both the ordered chromosomes (chr1-31, chrX, or MT collectively), and to chrUn. However, more than 85% of these short reads did not map to the ordered chromosomes. In addition, greater than 93% of them did not map to chrUn. Given these poor alignment percentages, mappings corresponding to these short reads were not included in the BAM file for variant analysis. Overall, our realignment procedure covered 96.2% of EquCab2 positions with at least three reads (average depth-of-coverage = 7.6).

**Table 1:** Summary of mapping statistics for the Twilight Sanger reads.

	Reads> = 200bases (28,183,476)	50 bases ≤ Reads<200bases (635,544)	
chr1-31 + chrX, chrMT	27,482,518 (97.5%)	89,652 (14.1%)	xxxxxx
chrUn	299,346 (1.1%)	xxxxxx	43,669 (6.9%)
Not mapped	401,612 (1.4%)	545,892 (85.9%)	591,875 (93.1%)

Mapping results can be viewed within IGV[9], or UCSC Genome Browsers[10]. Data available for inspection are the BAM files containing the mapped reads, VCF files which identify all positions in the mapped dataset that vary in the Twilight Sanger data, and in the Twilight Illumina data relative to the EquCab2 assembly, and a BED file that lists the regions in the genome with low Twilight Sanger coverage. These data may be accessed at http://dx.doi.org/10.13013/J6RN35SN (this link is also available in Table 2).

**Table 2:** DOI for the annotation data produced in this work.

URL to access annotation data	http://dx.doi.org/10.13013/J6RN35SN

### Coverage

**Fig 1:**
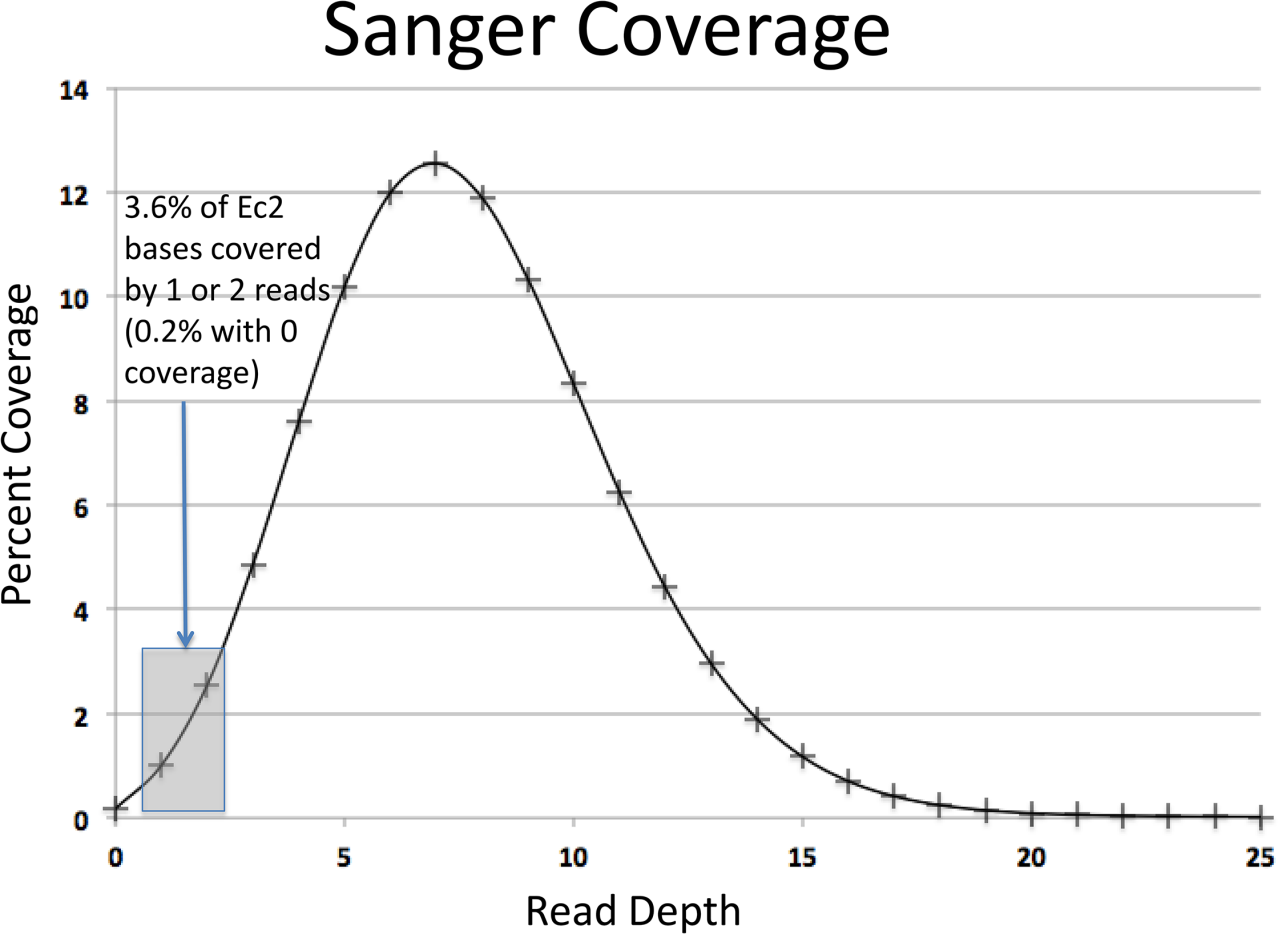
Depth of Sanger Read Coverage. A graph of depth of coverage for the mapped Sanger reads. The shaded gray box highlights the percentage of reads covered by one or two reads.

The coverage reported in the EquCab2 publication was 6.8X[1]. The coverage we were able to measure with the mapped reads was 7.6X. Excess coverage was due to a number of factors including the following: the BWA-SW program allows for multiple mappings of a read and the criteria for mapping a read are less stringent than for inclusion in an assembly. The coverage density is shown in Fig 1. One of the aims of this work was to provide a map of those bases within EquCab2 with coverage of two or fewer reads by the source Twilight and Bravo Sanger data. Nucleotides in the reference genome with low coverage would be more likely to have retained any errors found in the primary Twilight and Bravo Sanger reads. That BED file, indicating regions covered by two or fewer reads is also provided at the URL listed in Table 2. We identified 3.6% of the genome as having coverage of one or two reads. 0.2% of bases were found to have zero coverage. In a majority of cases, these zero coverage regions reflect trimming inconsistencies between our effort and that of Wade et al. (2009) at contig boundaries in the assembly flanked by single read coverage.

### Single nucleotide variants and small insertions or deletions that are inconsistent between Twilight and the reference

The haploid EquCab2 reference assembly was created using Sanger reads derived largely (99%) from a single individual. In the process, diploid alleles that reflect Twilight’s sire and dam were reduced to a haploid representation by retaining only one of the alleles for the reference genome. However, when sequence data from Twilight are mapped back to this reference, both alleles should be detectable. When performing variant discovery using the mapped source data, each position with two alleles should result in a heterozygous genotype call. In contrast, homozygous variants would represent either errors in the reference (i.e. the reference allele was an error present in the source data) or contributions from other animals.

We measured single nucleotide variants (SNVs) and small insertions or deletions (In/Dels) via the GATK UnifiedGenotyper[11] in three mapped datasets. The first was comprised of only the original Twilight Sanger reads (7.6X), a second was comprised of new Illumina paired end sequence data (~40X) also derived from Twilight, and the third dataset was the Bravo BAC end Sanger sequences. A total of more than 3.2 million heterozygous variants were detected in Twilight’s mapped Illumina dataset, which is much higher than the number of variants detected using the original Sanger Sequence data alone. The additional depth of coverage is the primary reason that the number of variants detected is higher in the current study relative to Wade et al (2009). The higher level of coverage greatly increases the likelihood that a second allele, if present, is detected. These results are shown in Tables 3 and 4.

**Table 3:** Variation identified in mapped Twilight Sanger data set.

Total Variants	1,901,342
Total Called Heterozygotes	1,817,807 (95.61% of Total)
Total Called Homozygotes	83,535 (4.39% of Total)

**Table 4:** Variation identified in mapped Twilight Illumina data set.

Total Variants	3,961,813
Total Called Heterozygotes	3,241,330 (81.81% of Total)
Total Called Homozygotes	720,843 (18.19% of Total)

From the same experiment, the percentages of measured homozygous variants were 4.39% in the Sanger dataset and 18.19% in the Illumina reads. As stated above, one explanation for homozygous differences between Twilight and the EquCab2 reference is that errors present in some of the original Sanger reads were selected for incorporation into the reference. The problem was exacerbated when the assembler software had to derive the reference sequence from areas of the genome with low read coverage. Our re-analysis the Sanger reads from Twilight identified 83,535 homozygous variants (Table 3). In contrast, using the new Illumina dataset, 720,843 called homozygous variants were detected (Table 4). This dramatic increase primarily reflects the fact that errors (either in base calling or in the original analysis of Twilight’s Sanger reads) were not present in the new Illumina data.

**Fig 2:**
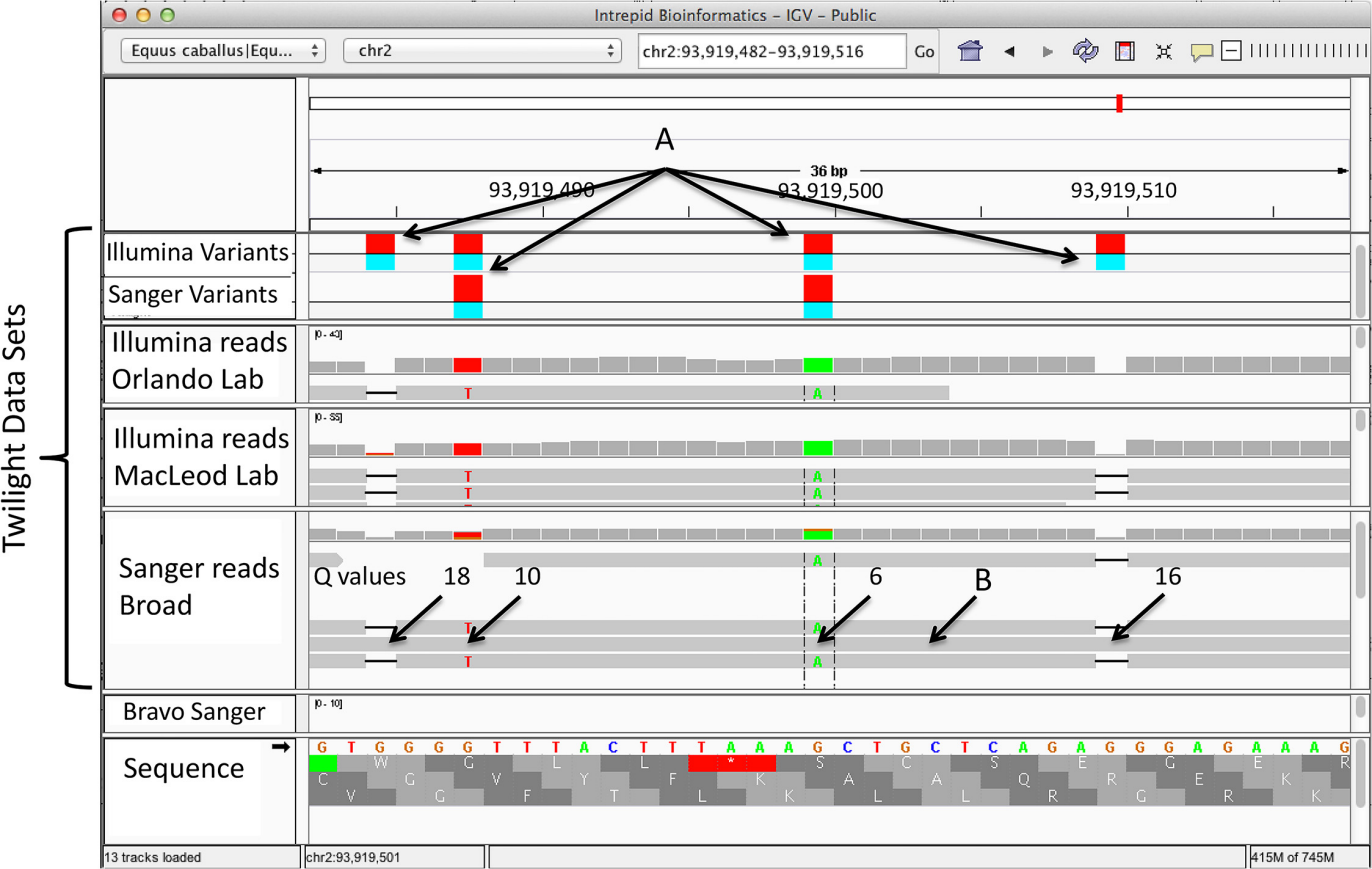
Incorrect Base Assignments. An example of incorrect base assignments. A) Variants (indicated by the red shading) that are called homozygous (indicated by the turquois shading) differences measured by the UnifiedGenotyper in both the Illumina and Sanger datasets are shown here. In B), it is demonstrated that a single low quality Sanger read was used as the basis for the consensus sequence in this region. The UnifiedGenotyper, however, ignores this read due to the low phred quality scores in the region. The phred based quality scores are indicated for the 4 miscalled bases. The corresponding NCBI Trace Archive Trace Name and TI# are G836P5757RI23 and 1325049864 respectively, with bases 594–630 as the region of interest. This region may be viewed in IGV at http://dx.doi.org/10.13013/J6VD6WCM. The link will download a JNLP file that webstarts IGV centered on region of interest. Bases within the reads that agree with the reference are not rendered.

For clarity, we describe how the terms homozygous/heterozygous difference versus a *called* homozygous/heterozygous difference are used. If Twilight differs from the reference genome in a homozygous fashion, the alleles actually present on her maternally and paternally inherited chromosomes are identical but differ from the allele reported in the reference. In some cases, the problem resulted from base calling errors in low quality Sanger reads. An example of this scenario is shown in Fig 2. It illustrates 4 positions, two insertions and two substitutions, that differ from all other reads collected for Twilight. The phred quality scores of those bases are all 18 or lower, clearly showing that the reference alleles were derived from a single low quality read. These sequencing errors, *called* differences, are responsible for the majority of the homozygous calls presented in Tables 3 and 4.

**Fig 3:**
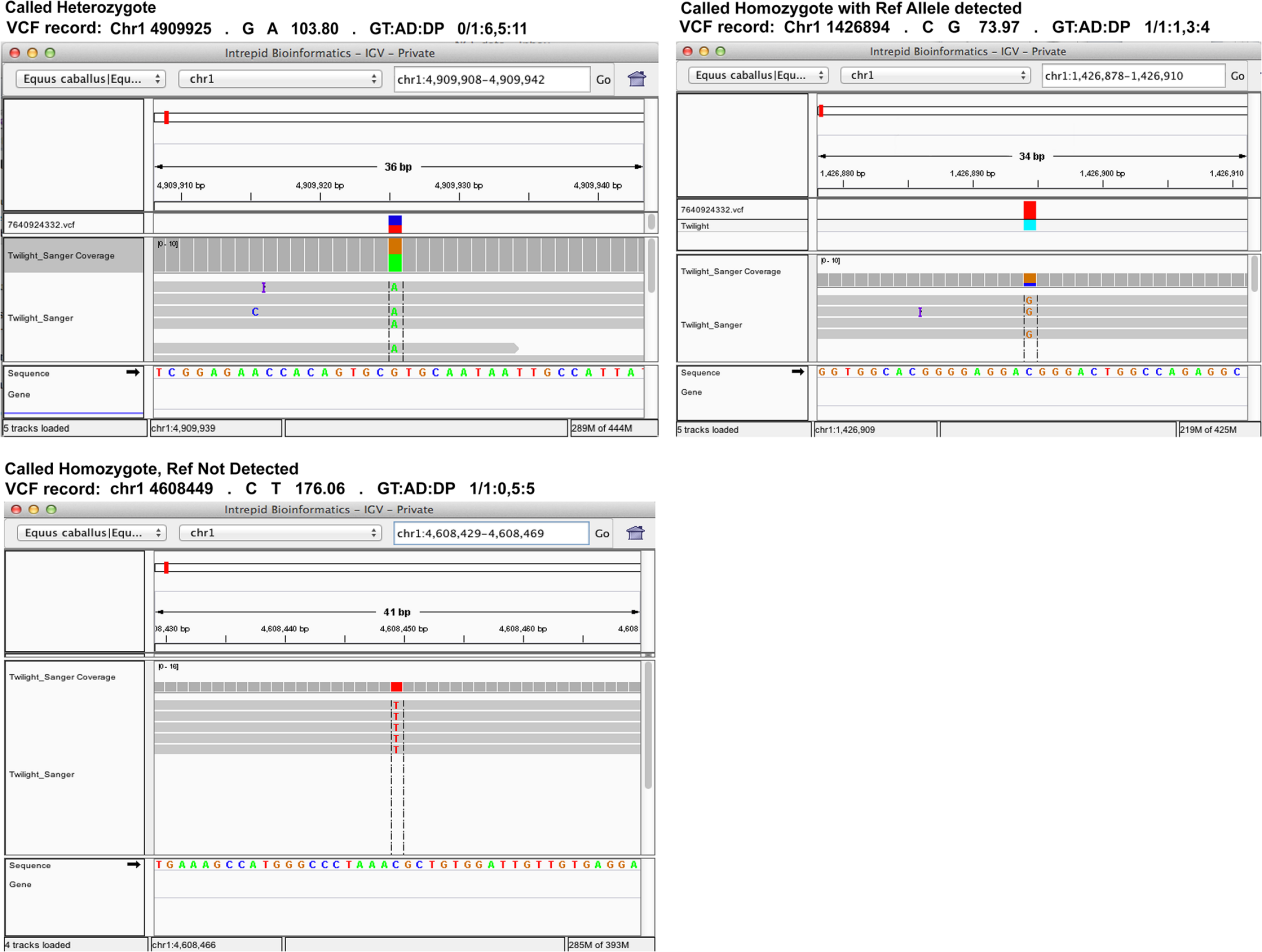
Variant Call Format Records. An example of three variant call format records. The first is a called heterozygote with an allele depth of 11, 6 reads containing the reference allele, and 5 reads containing the non-reference. The second, a called homozygote in the current analysis with an allele depth of 4, three high quality reads containing the non-reference allele and one low quality read containing the reference. The third, is an example of a called homozygote with an allele depth of 5, where all 5 reads contained the non-reference allele. Images of the reads used to derive the VCF records are shown within the IGV Browser. Base calls within the reads that agree with the reference are not rendered.

**Fig 4:**
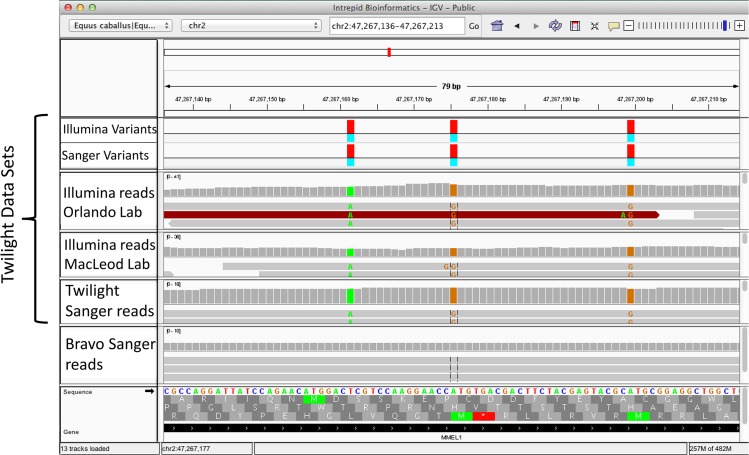
Twilight discrepancies resolved by Bravo data. An example of a region showing homozygous differences between Twilight and the reference which are resolved by alleles from Bravo. Region may be viewed in IGV at http://dx.doi.org/10.13013/J63X84K9. Link will download a JNLP file that will webstart IGV, centered on region of interest. Bases within the reads that agree with the reference are not rendered.

A second explanation is that the allele present in the reference was actually derived from genomic data contributed by a different horse. In this case, the BAC end sequence data from Twilight’s half brother named Bravo. To explore this possibility, we analyzed the “allelic depth (AD)” field from the VCF file. A detailed description of the VCF format can be found at http://vcftools.sourceforge.net/specs.html. Using the allelic depth field, we counted the number of homozygous variant positions (the 83,535 and 720,843 variant positions from the Sanger and Illumina data) at which the reference allele was not measured on any Twilight read (see Fig 3, “Called Homozygote, Ref Not Detected). This number would reflect the count of alleles potentially contributed by another animal. The subset of the 83,535 homozygous measured variants in the Sanger dataset where the reference allele was not detected on any read was 29,587 (Table 5). These may in part reflect a contribution of Bravo’s DNA to the EquCab2 reference. To test this, we genotyped Bravo for these positions in EquCab2. The results indicate that, for the Sanger data set, on the order of one third (9,828 out of the 29,587) of those positions queried in Bravo produced alleles that were consistent with the EquCab2 reference sequence (Table 5). An example of variants explained by the Bravo sequence are shown in Fig 4. The remaining 19,759 homozygous differences(29,587–9,828) cannot readily be attributed to either Bravo or Twilight. This may reflect our independent trimming and more stringent filtering of low quality Sanger reads or possibly an artifact of the assembly process, for example contributions from low quality alignments that would not map to the finished assembly.

**Table 5:** Called homozygous variants for which the reference was not detected in Twilight.

Variants	Sanger
Twilight homozygotes with no evidence of reference allele in Twilight	29,587
Reference attributable to Bravo	9,828

### Mapped dataset

The Sanger reads used in this work were pairs produced by sequencing the ends of inserts 4,000, 10,000, and 40,000 bases in length. However, the BWA-SW alignment program treated all reads in the Sanger dataset as independent, and created a BAM file with no pair information. Since mapping distances between corresponding Forward and Reverse reads from the same template provide useful information as to the presence of large insertions and deletions or genomic segments unaccounted for in the reference, we regenerated the BAM file and modified the bitwise flag and mate information fields to reflect this information.

## Discussion and Conclusion

Whether resequencing a whole genome, targeted genome regions, or transcriptome, the sequence data produced is traditionally mapped to the annotated reference genome appropriate for the species. Comparative analyses are then used to identify SNVs, small in/dels, structural rearrangements, or to quantify the expression of known or novel transcripts. The validity of this process is directly related to the accuracy of the reference genome. EquCab2 has demonstrated itself a very robust resource for these purposes.

Nonetheless, artifactual errors and mis-called bases are anticipated in a reference assembly. As large quantities of new equine sequencing data have been generated and mapped to EquCab2, anomalies have been identified. Such differences are often due to rare variants found in the reference animal; however, it should also be considered that the anomaly might be due to an assembly artifact or other error in the reference sequence. Without access to sequence data from the reference animal, it is nearly impossible to gauge the origin of observed discrepancies. Mapping of the Twilight and Bravo Sanger reads identified 0.2% of the reference genome not covered by a single read from the datasets. In total, an additional 3.6% of the bases in EquCab2 were covered by only one or two reads, which hinders the assembler deriving an accurate consensus in those regions. Greater coverage would have increased the representation of the correct allele. Sanger sequencing, although accurate, does have limitations, as the quality is low in the first 50 or so bases of the read and potentially the last several hundred bases of the read. End trimming, based on the quality of bases near the start and end of the read is commonly used, as was the case for the assembly of EquCab2 and in this work. The method used in both instances was a sliding window of 20 bases moving from the beginning of the read forward and from the end of the read backward. A base quality threshold was reached when the bases within the window had an average phred quality score of 20 or higher, indicating the potential for an error rate of equal to or less than 1 miscall in 100 calls. At this threshold level, there would be a 40% opportunity for an error in the sequence data in the 20 base windows. The Sanger method also has difficulty accurately determining the length of homopolymers. Obviously, the lower the read coverage in a given region, the more difficult it is to compensate for these shortcomings.

In this work, we have identified inconsistencies between the EquCab2 reference assembly and the source data that was used to build it. Specifically, with the inclusion of a new high coverage Illumina dataset, 720,843 called homozygous differences exist between Twilight and the reference assembly. This same dataset revealed a total of 3,241,330 positions at which two alleles were detected for Twilight. This number is much larger than that identified by Wade et al. (2009) due in large part to the increased coverage (40X vs. 6.8X). In addition, even though the horse reference genome is frequently referred to as “The Twilight Assembly”, approximately 10,000 homozygous nucleotide variants between Twilight and EquCab2 were identified that reflect the use of Bravo BAC-end reads in the original dataset. For the remainder of the homozygous differences between Twilight and the reference, the majority represents errant alleles with no apparent basis in the Twilight or Bravo. In addition to these differences, another result of this work is annotation for EquCab2 that will allow scientists to more objectively evaluate observed sequence differences between experimental samples and the equine reference genome. To assist in that endeavor, we have published BAM files of the Sanger reads featured in this study, as well as two other mapped datasets each comprised of approximately 20X coverage of short-read genomic sequence data derived from Twilight. These files may be loaded into the Integrative Genomics Viewer (IGV), UCSC, or other tools capable of interpreting BAM file data for comparison against sequence data generated by independent researchers for the investigation of regions of interest or ambiguities in their work. Another resource generated by the current study is a derivation of the number of Sanger reads that went into producing the consensus base at each position in EquCab2. The regions detailed in the BED files will serve as a roadmap of areas that would clearly benefit from additional sequencing of Twilight which may not only resolve single base errors, but could ultimately result in the placement of some of the contigs found in chrUn.

## Materials and Methods

### Data download

#### Twilight trace data download

All Twilight Sanger reads for this work were originally generated at the Broad Institute for the equine genome sequencing effort reported by Wade et al (2009), and were deposited in the TraceArchive at NCBI with the associated tags SPECIES_CODE = ‘EQUUS CABALLUS’, CENTER_NAME = ‘BI’, and CENTER_PROJECT = ‘G836’. A search of the NCBI trace archive using these search terms returns a count of 30,111,484 records. The perl script, query_tracedb was downloaded from the NCBI website and a command line script was created to download the data in 30,000 read “pages” of data.

(echo-n "retrieve_tar_gz scf 0b";./query_tracedb "query page_size 30000 page_number XXX binary SPECIES_CODE = 'EQUUS CABALLUS' and CENTER_NAME = 'BI' and CENTER_PROJECT = 'G836‴) |./query_tracedb > dataXXX.tgz

The above script was replicated for each of the 1004 pages, where the XXX was replaced with the appropriate page number (page 0–1003). One thousand or fewer of these 1004 commands were aggregated in 11 shell scripts (4 commands in the 11^th^ script). Each command produced a corresponding tgz file, each containing as many as 30,000 scf files. Not all of the original records had a corresponding scf file. Ultimately, of the 30,111,484 total records, we were able to retrieve scf data for 30,107,623. No more than three of the larger shell scripts were run concurrently, and the download took approximately three weeks to complete.

In a similar fashion, the annotation information for all reads was downloaded with the script

(echo-n "retrieve_gz info 0b";./query_tracedb "query page_size 30000 page_number XXX binary SPECIES_CODE = 'EQUUS CABALLUS' and CENTER_NAME = 'BI' and CENTER_PROJECT = 'G836‴) |./query_tracedb > dataXXX.tgz;

Information contained within the “Information” file for each read included the vector trim positions for both the start and the end of the read.

#### Bravo trace data download

The Bravo reads were downloaded as above using the command

(echo-n "retrieve_tar_gz scf 0b";./query_tracedb "query page_size 40000 page_number 0 binary CENTER_NAME = 'GBF' and SPECIES_CODE = 'EQUUS CABALLUS' and INSERT_SIZE = 171000") |./query_tracedb > bravoXXX.tgz

### SCF file processing

An initial check of the annotation embedded in the scf files was performed by using the phred basecaller with the–nocall option to produce phd files. This revealed that the Life Technologies KB Basecaller was used to do both the trace processing and to derive the basecalls and quality scores embedded in the scf file. As the KB Basecaller has been reported to be more accurate on reads generated via an ABI 3730[12], we chose to use those embedded base calls and quality scores for our alignment work. As such, the–nocall option was used with the phred basecaller in order to produce phd files that contained, in addition to annotation information, three numeric columns which held the basecall, the quality score, and the pixel position of the corresponding peak, respectively.

### Quality and vector trimming

For the Twilight reads, the position used for quality trimming was identified by first creating an ordered integer array of the quality scores for the read. A sliding window of 20 quality scores was then moved from the beginning of the array inward. Once the sum of the quality scores within the window reached 400 (average of 20) or greater, the first position of the window was used as the left quality index. The right quality index was calculated in the same manner by placing the sliding window at the far end of the array of quality scores and moving it inward. The left and right vector trim positions were then extracted from the companion information downloaded for the reads. The position ultimately used to trim the left hand side was the greater of the left quality index, or the end position of the left vector. The right trim position was calculated by taking the smaller of the right quality index, or the start position of the right vector. The trimmed Twilight reads were then rendered in fastq format for mapping. The Bravo reads were converted from fasta/qual format to fastq, and were subsequently mapped without quality or vector trimming.

### Read mapping

BWA has within it a Smith-Watterman Aligner (BWA-SW) that was developed specifically to handle longer (> = 200 bases) reads such as those produced in a Sanger run The module was used in conjunction with the EquCab2 reference assembly, excluding the unincorporated contigs from chrUn to produce an unsorted SAM file. These reads were subsequently sorted, converted to BAM files, and merged using SAMtools[13]. Reads that did not map to the autosomal chromosomes 1–31, chrX, or MT were then identified and extracted to a separate fastq file for mapping to chrUn. Reads less than 50 bases in length after trimming were discarded. The reads whose trimmed length was less than 200 bases and greater than or equal to 50 were mapped using the standard BWA pipeline which includes generating an aln file, and subsequently reprocessing to create a SAM file. For reasons described in Results, the reads less than 200 bases and greater than or equal to 50 in length were ultimately not included in the mapped dataset used for variant analysis.

The Bravo reads were mapped using BWA-SW, and soft trimmed at the ends by the BWA algorithm.

### Illumina sequencing, MacLeod lab

The sequencing performed by the MacLeod lab was done with the following protocol. Genomic DNA was sheared using a Covaris Disruptor (http://covarisinc.com/). A gDNA fragment library was prepared using Illumina bead based size selection with an average insert size of 300–400 bp according to the manufacturer’s protocol (http://support.illumina.com/sequencing/sequencing_kits/genomic_dna_sample_prep_kit.ilmn). Paired end sequencing of the library was performed on an Illumina HiSeq 2000, 100 cycles.

### Trimming and filtering, MacLeod lab

Genomic DNA sequencing reads were first filtered by quality score using the FASTX-toolkit (version 0.0.13). Only reads with at least 50 percent of bases having a quality score of 30 or higher were retained. Two trimming steps were then applied. First, starting at the 3’ end, any bases with a quality score less than 3 were removed until a nucleotide that met this threshold was reached (FASTX-toolkit, version 0.0.13). Then, any Illumina TruSeq adapter sequences were removed from the reads (Cutadapt, version 1.2.1). Finally, after these quality trimming and adapter clipping steps were completed, only reads with a total length of 50bp or greater were retained. A custom script was used to synchronize the two ends of the reads.

### Illumina sequencing and trimming, Orlando lab

The process for sample prep and subsequent processing of read data is described in Orlando et al.[5]

The Illumina reads generated by the MacLeod and Orlando labs are deposited in, and available through the NCBI Sequence Read Archive, accessions SRR901148, SRR901233, SRR901234, SRR901235, SRR1055837

### Variants called with UnifiedGenotyper

The BAM files produced using BWA were analyzed for variation using the GenomeAnalysisToolkit’s UnifiedGenotyper with default parameters.

### Coverage calculation

The coverage histogram shown in Fig 1 was derived by first processing the BAM file created by merging the Twilight and Bravo BAM files and then producing a pileup format file via SAMtools. The depth at each position was derived from the number of reads represented at each base. Bases with zero coverage were not represented in the pileup file. In the majority of the cases, base positions were run sequentially, however occasional gaps indicated base positions that had zero coverage. To derive the number of bases with zero coverage, the value for the left flank of the gap was subtracted from the value of the right flank of the gap and one was subtracted. This value was added to the zero bin of the histogram. Since many of these gaps were the result of an arbitrary number of N’s used to indicate contig boundaries, the number of bases with zero coverage was initially overestimated. The number of N’s in EquCab2 was calculated by creating a modified version of the reference with the N’s removed. Lengths of the resulting new chromosomes were determined and subtracted from the length of the uncorrected chromosome sequence in EquCab2. This corrected number was used as the value for the zero coverage bin in Fig 1. To derive the fold coverage value, the number of bases with a specific depth (y axis in Fig 1) was multiplied by that depth (x axis in Fig 1) for each depth value considered (x values 1 to 25). We did not consider depths greater than 25 in this calculation, as anything greater was likely the result of inflated coverage due to a repeat region.

### SAM file reprocessing

As described above, the Twilight Sanger data was mapped to the EquCab2 reference genome, each as an independent read. However, in nearly every case, each read had a corresponding mate produced by sequencing the opposite end of the template. These were treated in a BAM file analogously to paired end reads. The fields in the SAM file include the Mate Reference sequence NaMe (MRNM), 1-based Mate POSition (MPOS), and Inferred insert SIZE (ISIZE). These, and the bitwise FLAG (FLAG) fields all may be set with useful information. However, the initial BAM file created by BWA-SW contains none of this information. The FLAG was set at 0 for a read mapped in the forward orientation relative to the reference, 16 for a read mapped in the reverse orientation relative to the reference, and 4 if the read is unmapped. There were a small number of clones that were sequenced multiple times in either the forward or reverse direction relative to the clone. In these cases, we defined an exemplar read for that direction/clone by selecting the single read for that direction/clone which was greater than 50 bases long and had the highest average quality score. The non-exemplar reads are labeled via the FLAG as “optical or PCR duplicates” (+1024). For all reads that had a mate used in the study, 1 was added to the FLAG. If the mate was used but unmapped, 8 was added to the FLAG. The read direction was encoded in the read name. Those reads with an “F” were treated as the “first read in pair”, adding 64 to the FLAG. Those with an “R” were treated as the “second read in pair”, adding 128 to the FLAG. For those reads that were mapped multiple times, the read with the highest mapping quality greater than 40 was taken as the primary alignment. All other SAM records had 256 added to them indicating that they are not the primary alignments. For these cases of multiple alignments, the mapping position of the mate’s primary alignment was used for the corresponding MRNM, MPOS, and ISIZE fields.
